# The Suicidal Physician: When the Doctor Wants to Die

**DOI:** 10.1192/j.eurpsy.2023.2358

**Published:** 2023-07-19

**Authors:** D. O. Martins, M. Pinho, P. S. Martins, L. Gomes, E. G. Pereira

**Affiliations:** 1 Hospital de Magalhães Lemos, EPE, Porto, Portugal

## Abstract

**Introduction:**

Medical-related professions are at high suicide risk. Suicide is a major cause of premature death among physicians, but the prevalence of suicide-related behaviors is inconsistent across studies.

**Objectives:**

Presenting a review of the prevalence and risk factors of suicide among physicians.

**Methods:**

Search on Pubmed® and Medscape® databases with the following keywords: “physicians” and “suicide”. We focused on data from systematic reviews and meta-analyzes. The articles were selected by the authors according to their relevance.

**Results:**

Female and US physicians were at higher risk of suicidal behavior. Suicide decreased over time, especially in Europe. Some specialties might be at higher risk such as anesthesiologists, psychiatrists, general practitioners and general surgeons. It is well established that anesthesiologists tend to have much higher rates of substance abuse than other physicians. Psychiatrists are also known to have more mental distress, mental illness and burnout compared with other physician groups and have concerning rates of depression and psychotropic. Physicians are less likely to seek mental health services out of career concerns, culture and/ or a predisposition toward self-reliance. Additionally, retrospective toxicology screening of suicide data finds that physicians are more likely than nonphysicians to have positive results for antipsychotics, benzodiazepines, and barbiturates but not antidepressants.

**Conclusions:**

Physicians are an at-risk profession of suicide, with women particularly at risk. The rate of suicide in physicians decreased over time, especially in Europe. The high prevalence of physicians who committed suicide attempt as well as those with suicidal ideation should benefits for preventive strategies at the workplace. Physician suicides are multifactorial, and further research into these factors is critical. Appropriate preventive and treatment measures should be implemented to reduce the risk of suicide-related behaviors in this population.

**Disclosure of Interest:**

None Declared

